# Systems Pharmacology Dissecting Holistic Medicine for Treatment of Complex Diseases: An Example Using Cardiocerebrovascular Diseases Treated by TCM

**DOI:** 10.1155/2015/980190

**Published:** 2015-05-26

**Authors:** Yonghua Wang, Chunli Zheng, Chao Huang, Yan Li, Xuetong Chen, Ziyin Wu, Zhenzhong Wang, Wei Xiao, Boli Zhang

**Affiliations:** ^1^Evidence-Based Medicine Centre, Tianjin University of Traditional Chinese Medicine, The First Affiliated Hospital, Tianjin University of Traditional Chinese Medicine, Tianjin 300193, China; ^2^Bioinformatics Center, College of Life Sciences, Northwest A&F University, Yangling, Shaanxi 712100, China; ^3^School of Chemical Engineering, Dalian University of Technology, Dalian 116024, China; ^4^State Key Laboratory of New-Tech for Chinese Medicine Pharmaceutical Process, Lianyungang, Jiangsu 22001, China

## Abstract

Holistic medicine is an interdisciplinary field of study that integrates all types of biological information (protein, small molecules, tissues, organs, external environmental signals, etc.) to lead to predictive and actionable models for health care and disease treatment. Despite the global and integrative character of this discipline, a comprehensive picture of holistic medicine for the treatment of complex diseases is still lacking. In this study, we develop a novel systems pharmacology approach to dissect holistic medicine in treating cardiocerebrovascular diseases (CCDs) by TCM (traditional Chinese medicine). Firstly, by applying the TCM active ingredients screened out by a systems-ADME process, we explored and experimentalized the signed drug-target interactions for revealing the pharmacological actions of drugs at a molecule level. Then, at a/an tissue/organ level, the drug therapeutic mechanisms were further investigated by a target-organ location method. Finally, a translational integrating pathway approach was applied to extract the diseases-therapeutic modules for understanding the complex disease and its therapy at systems level. For the first time, the feature of the drug-target-pathway-organ-cooperations for treatment of multiple organ diseases in holistic medicine was revealed, facilitating the development of novel treatment paradigm for complex diseases in the future.

## 1. Introduction

Holistic medicine is a system of health care which focuses on the whole person rather than just on the illness or part of the body that is not healthy [1, 2]. The concept of holistic medicine can trace back to the 4th century B.C. [3]. Since then, considerable knowledge has been accumulated concerning its efficacy and safety in treating ill health. However, from the beginning of the twentieth century, the principle of holistic medicine has been gradually falling out of favor in Western societies, with the enormous advancements in what we now call “allopathic” medicine. Paradoxically, the increasing evidence in clinic shows the allopathic medicine has got into trouble for handling complex diseases, particularly, the chronic diseases, as it mainly focused on particular body components to treat. In this case, the nonreductionist systems medicine [4], which draws on theories from holistic medicine, has been proposed to consider complex interactions within the human body to promote an individual's health in light of their genomics, behavior, and the external environment.

The complex diseases, such as tumors, diabetes, cardiocerebrovascular diseases (CCDs), infectious diseases, and many more, largely arise from an integration of genetic, environmental, and lifestyle factors [5]. A notable example is the cardiorenal syndrome (CRS) [6], which is employed for the coexistence of heart and kidney malfunction. And so far, there is still no effective cure in allopathic medicine to head off the process from cardiovascular diseases (CVDs) to kidney disease. A major obstacle to complex disease treatment is that the present one drug-one target-one disease philosophy is incapable of dealing with the complex nature of these illnesses. Thus, people are now returning to holistic medicine to find newfangled therapeutics aiming at multiple targets/organs interactions for preventing and treating of diseases.

There are some health-prevention systems that come under the umbrella of holistic medicine, especially the traditional Chinese medicine (TCM). In contrast to allopathic medicine, TCM seeks to treat the body as a whole, which fosters a cooperative relationship among all elements (molecule, cell, tissue, organ, etc.) involved, leading towards optimal health and wellness. Under the holistic concept, in clinic practice, a large number of effective TCM prescriptions for treating various diseases have been developed since 2,000 years ago, for example, compound Danshen formula [7], Shexiang Baoxin formula [8], Curcumae formula [9], and so forth. Although the concept of holistic medicine has been revealed to some extent in TCM practice, scientific evidence to illustrate the mechanisms of holistic medicine is severely lacking and many key problems associated still exist: for example, (1) in TCM framework, how the body correlates with the external environment and adjusts to it; (2) from molecules to organs, how the body responds to drug treatment and returns to a normal dynamic balance; (3) how the herb system (active ingredients) interacts with the body system, resulting in curing of disease.

In an attempt to uncover the holistic way of TCM from a molecular to systems level, many researchers have done plenty of constructive work over the past years [10, 11]. Representative of typical technologies are chemical [12], pharmacokinetic [9], and pharmacological [13] approaches. These works have, respectively, tended to focus on component identification, drug metabolism, and drug action mechanism investigation when discussing pathological conditions, rather than focusing on the foremost holistic feature of TCM, despite the importance of the latter in physiological phenomena. However, such identified active compounds in TCM may provide clues as to their potential for addressing multiple targets* in silico*, such as the omic-based ligand-target chemogenomic model (LTC) [14]. Furthermore, mapping of the screened compounds onto their associated biological pathway may suggest potential sites of interaction between TCM-derived ingredients and human body. Thus, the integration of the above technologies will provide deep insights into data consolidation and analysis associated with TCM. Once constructed, it will be possible to deconstruct the molecular pathways and open new horizons for disease management, for example, by linking the genes expression levels and their respective targets with the administered TCM active ingredients/metabolites. Such technique will no doubt be favorable for the modernization of TCM and the establishment of the multicomponent, multitargeting strategy as a new paradigm in medicine.

In this study, we develop novel systems pharmacology approach to dissect holistic medicine in treating complex diseases by TCM, which is exemplified by CCDs. Currently, CCDs remain the major cause of death in the United States and constitute 17% of overall national health expenditures [15] due to the inefficiency of the present world's most advanced molecular targeted therapies in clinical practice. However, the successful applications of TCM in CCDs [7, 9] bring hope to these patients who are trapped into systemic symptoms caused by gene-organ-environment interactions. Interestingly, the pathogenesis of CCDs is consistent with the holistic concept of TCM. To decode the role TCM plays in treating complex diseases, the following steps are proposed: firstly, by applying the TCM active ingredients as baits, we fished and experimentalized the signed drug-target interactions to evaluate the pharmacological actions of drugs at a molecule level; then, at an organ level, we focused on exploring the tissue distribution of targets by a gene extraction method; finally, the translational integrating pathway approach was applied to construct complex diseases therapeutic modules for understanding the mechanism of complex diseases at systems level. As we have seen, this is the first time to illustrate the nature of that holistic medicine which provides effective access to prevention and cure of complex diseases. We believe that holistic medicine of investigating a molecule to systems level therapeutic of disease will facilitate the development of new treatment paradigms for complex diseases in the future.

## 2. Materials and Methods

**Figure 1:**
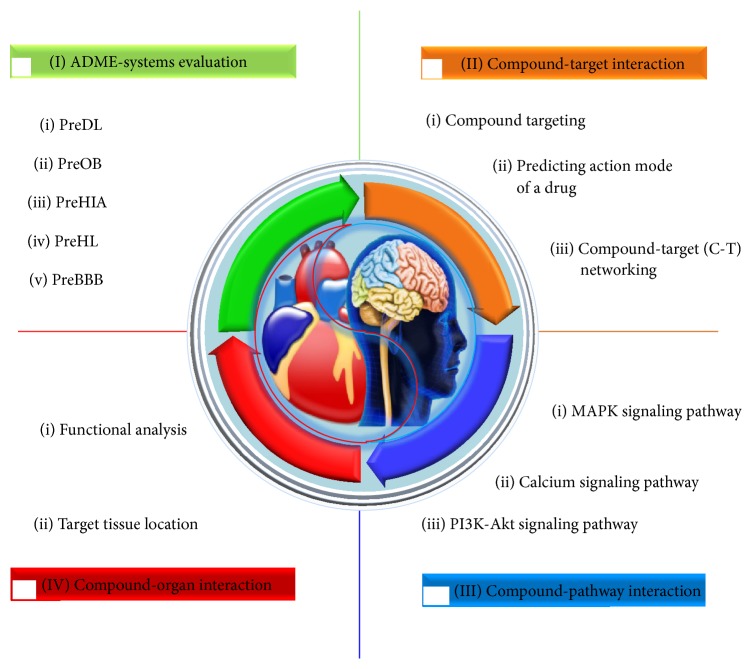
Workflow for holistic medicine in the treatment of complex diseases.

In this section, we propose a novel systems-based approach which is composed of four modules to explore the nature of holistic medicine (Figure 1). Specifically, we adopt an orally administered Chinese patent medicine Xinnaoxin Pill (Table S1 in Supplementary Material available online at http://dx.doi.org/10.1155/2015/980190) consists of* Rhodiola rosea* (*R. rosea*),* Lycium chinense* Miller (*L. chinense*), and* Hippophae rhamnoides* Linn. (*H. rhamnoides*) that has been applied in clinic to treat CCDs, as an example to illustrate the proposed model.

### 2.1. ADME-Systems Evaluation

**Figure 2:**
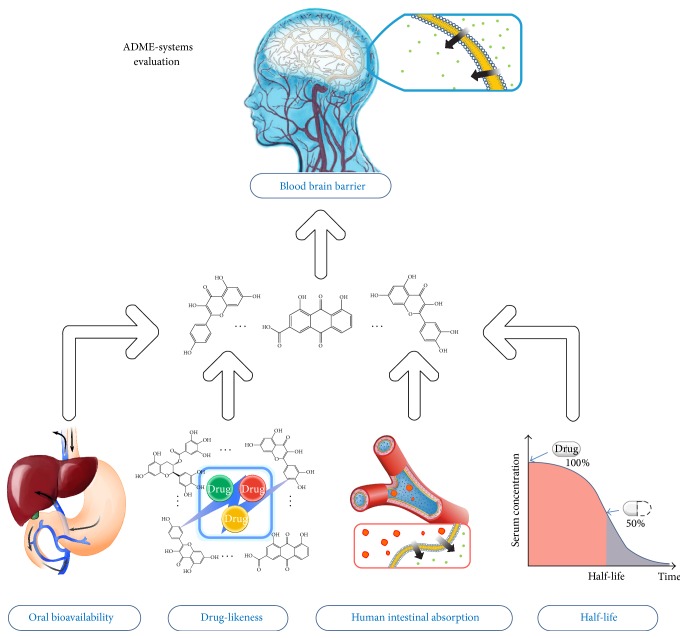
ADME-systems evaluation model.

An in house* in silico* ADME (absorption, distribution, metabolism, and elimination) integrative model is developed for evaluating the most important ADME properties of a drug molecule, that is, PreOB (predicts oral bioavailability), PreHIA (predicts human intestinal absorption), PreDL (predicts drug-likeness), and PreBBB (predicts blood-brain barrier) (Figure 2).

#### 2.1.1. PreOB

Owing to the fact that OB is one of the most important determinants of the dosing regimens for drugs [16], PreOB directs toward identifying final oral compounds in drug prescreening process. PreOB was supported by a dataset of 805 structurally diverse drugs with determination coefficient (*R* ^2^) of 0.80 and standard error of estimate (SEE) of 0.31, which brought P-glycoprotein (P-gp) and cytochrome P450s into the construction of the prediction model, which has been successfully applied in many drug screening studies [17].

#### 2.1.2. PreHIA

To predict human intestinal absorption for drugs, a new model is generated by means of the classification-partial least squares (C-PLS) method [18]. PreHIA mainly includes the following three steps. 

(*1) Data Sets.* A total of chemically dissimilar 732 compounds with their quantitative human intestinal absorption percentage values were collected from three studies [19–21]. The compounds are mainly absorbed by passive diffusion and without known issues such as dose-limited, dose-dependent, or formulation-dependent influence. The HIA values of drugs greater than 70% were deemed as the judging boundary for high absorption and low absorption. 

(*2) Descriptor Calculation and Selection.* To calculate representations of the structural and physicochemical features of the molecules [7], the DRAGON 5.4 program (http://www.talete.mi.it/index.htm) was applied on 929 2D molecular descriptors. Objective feature selection based on forward stepwise algorithm was then employed to discard redundancy and noncontributing descriptors in the descriptors pool and eventually 49 (Table S2) of them were obtained and further applied for C-PLS modeling process. 

(*3) Model Performance.* The dataset is separated into training and test sets by an internal 10-fold cross-validation. The accuracies for overall, high absorption, and low absorption prediction were used to measure the performance of the PLS model. As a result, the derived model shows impressive performance of prediction for HIA by 5 PLS components, with an overall accuracy of 83.7%, a high absorption prediction accuracy of 81.8%, and a low absorption prediction accuracy of 91.5% (see details in Figure S1). 

#### 2.1.3. PreDL

PreDL was developed to recognize molecules that are “drug-like” and capable of modulating targets, which has been successfully applied in many studies [9, 22]. The DL values were obtained by calculating the Tanimoto similarity [23] between herbal compounds and the average molecular properties of all compounds in the Drugbank database [24].

#### 2.1.4. PreBBB

To detect whether the biologically active compounds can pass through the blood-brain barrier, an updated model previously constructed by PLS algorithm is developed. PreBBB contains 190 related but chemically diverse compounds which are strong, moderate, or nonpenetrating cross the blood-brain barrier [25].

In this work, the threshold values for the integrative ADME screening system are OB ≥ 30%, DL ≥ 0.18, and HIA ≥ 70%. And the compounds with BBB < −0.3 were considered as nonpenetrating (BBB−), from −0.3 to +0.3 moderate penetrating (BBB±), and >0.3 strong penetrating (BBB+). Molecules that successfully meet 67% (2/3) of the criteria are nominated as bioactive compounds for further analysis.

### 2.2. Drug-Target Interaction

For the elucidation of the interactions between target proteins and all drugs, the quantitative analyses of drug targeting, action mode of a drug, drug-target (D-T) network, and* in vitro* experimentation were investigated.

#### 2.2.1. Drug Targeting

To obtain cerebrovascular diseases (CBVDs) and CVDs target profiles, a comprehensive drug targeting approach integrating the text mining, database search, and chemometric analysis was applied. First, a full-text data mining was carried out in TCMSP database (http://lsp.nwsuaf.edu.cn/tcmsp.php) to derive the molecular target information. Second, a chemical fingerprint-based Similarity Ensemble Approach was applied to obtain the potential targets (http://sea.bkslab.org/search/). Third, an in-house LTC [14] was further introduced for expanding the target pool. The targets from different sources were connected to database UniProt (http://www.uniprot.org/) for target name standardization, which were further subjected to PharmGkb [26], Therapeutic Target Database [27], and the Comparative Toxicogenomics Database [28] to delete noise and errors and to ensure the quality of target database.

#### 2.2.2. PreAM: Predicting Mode of Action for Drugs

In an effort to stringently assess the relationships between compounds and corresponding targets, for the first time, a new PreAM model was built in this work. The data sets of drug structures and protein sequences for drug-target interactions (DTIs) with known action modes were retrieved from DrugBank database (http://www.drugbank.ca/, accessed on October 1, 2013). Here, we separated these DTIs into two categories based on their action modes: (1) activated DTIs, where the description label covers “agonist,” “activator,” “inducer,” “stimulator,” and “partial agonist”; (2) inhibited DTIs, where the action label contains any of the keywords “inhibitor,” “antagonist,” “inactivator,” “negative modulator,” “partial antagonist,” “suppressor,” and “reducer actions.” In total, 6,006 DTIs (including 1,251 activated DTIs and 4,755 inhibited DTIs) were used as benchmark data (Table S3).

To characterize the interactions of drug and protein, drug structures and protein sequences were converted into numerical descriptors by employing DRAGON program (http://www.talete.mi.it/index.htm) and PROFEAT WEBSEVER (http://jing.cz3.nus.edu.sg/cgi-bin/prof/prof.cgi/), respectively (see details in Table S3). The multiple DTIs were represented by concatenating these chemical and protein descriptors and the minimal-redundancy-maximal-relevance (mRMR) was applied as a variable selection strategy to recognize the best combination descriptors that are most relevant to obtain the models with the highest predictive power. The first 100 descriptors were used in subsequent study.

The random forests (RF) algorithm (http://www.stat.berkeley.edu/users/breiman/) was trained to generate a nonlinear classifier tailored to DTIs with known action modes. The accuracies to overall, activation, inhibition were used to measure the performance of the model. The derived model shows impressive performance of prediction for drug-target interactions, with an overall accuracy of 97.3%, an activated prediction accuracy of 87.7%, an inhibited prediction accuracy of 99.8%. (see details in Figure S2).

#### 2.2.3. Drug-Target (D-T) Network

To characterize the multicomponent therapeutic features in the treatment of CBVDs and CVDs, a drug-target network is generated, in which a compound and a target are linked if a compound targets a known protein. The compounds are the ones which are screened out from the ADME systems, with their targets derived from the above targeting process. The bipartite graphs are visualized and analyzed by Cytoscape version 2.8.1 [29].

#### 2.2.4. Experimental Validation

To validate the practicability and efficiency of above methods, the inhibitory effects of drugs on predicted targets were quantified by ligand-binding assays according to the manufacturer's instructions. The drug-target interactions were selected at random and readily available on the market, respectively. Compounds rhodiosin, astragalin, rutin, quercetin, astragalin, and kaempferol-3-glucoside were from Yitai Technology Ltd. (Wuhan, China). Targets PIM1 and PTGS1 were respectively purchased from Cayman Chemical, Ann Arbor, MI, USA and CycLex, Japan. The purity of all the compounds is >98%. All drugs were dissolved in DMS and freshly prepared due to loss of activity under long-term storage. IC_50_ values were determined using the Bliss method with three independent determinations.

### 2.3. Drug-Pathway Interaction

To investigate the biological effects exerted on the pathway level, an incorporated “CCDs pathway” was assembled based on the current knowledge of CCDs pathology. First, the obtained target profiles were organized into several pathways by mapping them onto KEGG database (http://www.genome.jp/kegg/). Then, the pathways, which are not directly related to CCDs, were removed according to the pathological and clinical data. Finally, a relatively complete CCDs pathway was manually synthesized, including MAPK signaling pathway, calcium signaling pathway, and PI3K-Akt signaling pathway. By adopting the *φ*_*pp*′_ expression in HmSP [30], we further make a nearness analysis on the correlativity between the obtained targets and the “CCDs pathway” related proteins in the protein-protein interaction (PPI) network.

### 2.4. Drug-Organ Interaction

To understand the underlying connections of CBVDs and CVDs on organ level, it is essential to check the functional and tissue expression profile of the protein targets of the two related diseases. Firstly, we enrich the overrepresented gene ontology (GO) terms and check the expressed tissue distribution in the obtained target networks [31]. For GO analysis, the biological process of GO vocabulary (GOBP) was highlighted. The target tissue distribution was determined based on the microarray analyses data of different tissue types lodged in the BioGPS bank (accessible at http://biogps.org/), an extensible and customizable portal for querying and organizing gene annotation resources. The expressions for targets tissue distribution are as follows:

(1) $$\begin{array}{c} \Omega =\left\{\;t_{1},t_{2},\ldots ,t_{n}\;\right\}, \end{array}$$

where *t* is the human body organization and *Ω* is the set of tissues:

(2) $$\begin{array}{c} H_{i}=\left(\;h_{it_{1}},h_{it_{2}},\ldots ,h_{it_{n}}\;\right), \end{array}$$

where *h* is the tissue-specific pattern of mRNA expression of a target and *H*_*i*_ is the mRNA expression set of a target in *Ω*:

(3) $$\begin{array}{c} \bar{h_{i}}=\frac{\sum_{i=1}^{n}h_{i}}{n}, \end{array}$$

where $\bar{h_{i}}$ is the average expression quantity of a target in tissues and *n* is the number of tissues:

(4) $$\begin{array}{c} A_{i}=\left\{\;t\in \Omega \;|\;t>\bar{h_{i}}\;\right\}, \end{array}$$

where *A*_*i*_ is the tissue localization of a target.

## 3. Results

All the compounds, biologically active compounds, and targets for Xinnaoxin Pill have been uploaded to our TCMSP database. All readers can freely access the TCMSP database.

### 3.1. Multicomponent Therapeutics

As shown in Table 1, 18 compounds in Xinnaoxin Pill match all the filter criteria in Section 2.1. There are 1, 5, and 9 compounds that exist only in* R. rosea*,* L. chinense*, and* H. rhamnoides*, respectively.

#### 3.1.1. *R. rosea*

The bioactive ingredients in* R. rosea* are kaempferol, quercetin dehydrate, rhodiosin, and rutin, which possess both therapeutic effects for CBVDs and CVDs. For kaempferol, it was reported that persons with high kaempferol intakes normally have low incidence of CBVDs [32]. Quercetin can prevent heart disease [33] and rhodiosin shows a strong antioxidant activity [34] and thus remedies coronary artery disease [35]. In addition, Harvard researchers recently found that rutin has potent anticlotting powers for preventing heart attack [10].

#### 3.1.2. *L. chinense*

The main active substances in this herb are glycitein, mutatoxanthin, quercetin, and so forth (Table 1). Glycitein is suggested to be a potential alternative to estrogen therapy in the treatment of CVDs [36]. Mutatoxanthin may be scavenger of reactive species, thus showing antioxidant activity [37], which is similar for quercetin cardioprotective activities supported by* in vitro* and animal studies [38, 39].

#### 3.1.3. *H. rhamnoides*

The potential active substances are astragalin, flavoxanthin, vitamin K, and so on (Table 1). Astragalin has positive myocardial inotropic effects by inhibiting the platelet aggregation [40]. Flavoxanthin shows preferable activity in reduction of degenerative heart disease [41]. Vitamin K plays a preventive role in coronary artery calcification for the properties of matrix Gla protein.

Of the 18 bioactive compounds in Xinnaoxin Pill, eight compounds can freely cross the BBB (Table 1) and act as the main therapeutics for the treatment of CBVDs. The other BBB nonpermeable molecules are considered to combat CVDs.

### 3.2. Drug-Target Interaction

**Figure 3:**
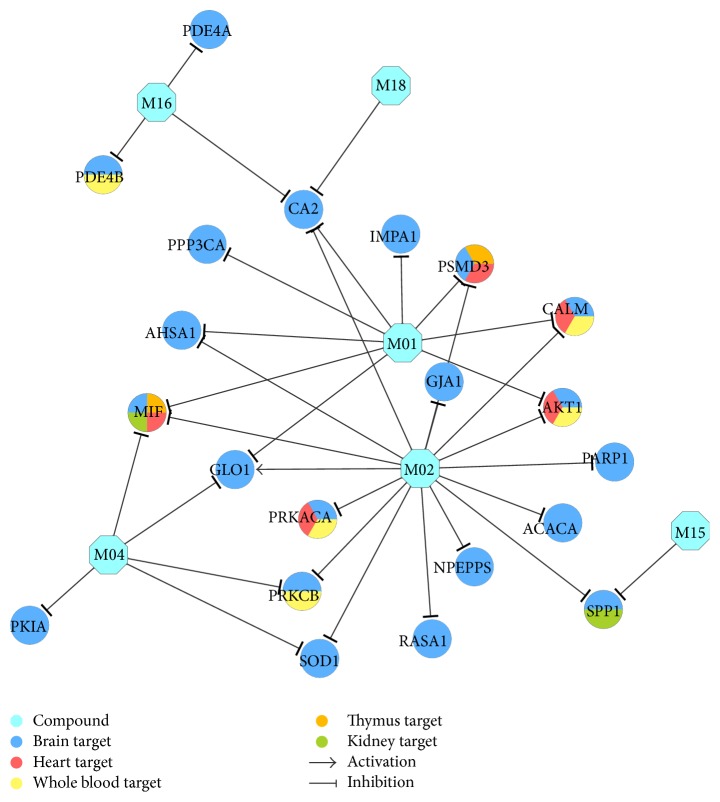
CBVDs therapeutic compound-target network. A compound node and a protein node are linked if the protein is targeted by the corresponding compound. Node size is proportional to its degree. The letters are target labels. Arrows indicate activation and T-arrows represent inhibition.

**Figure 4:**
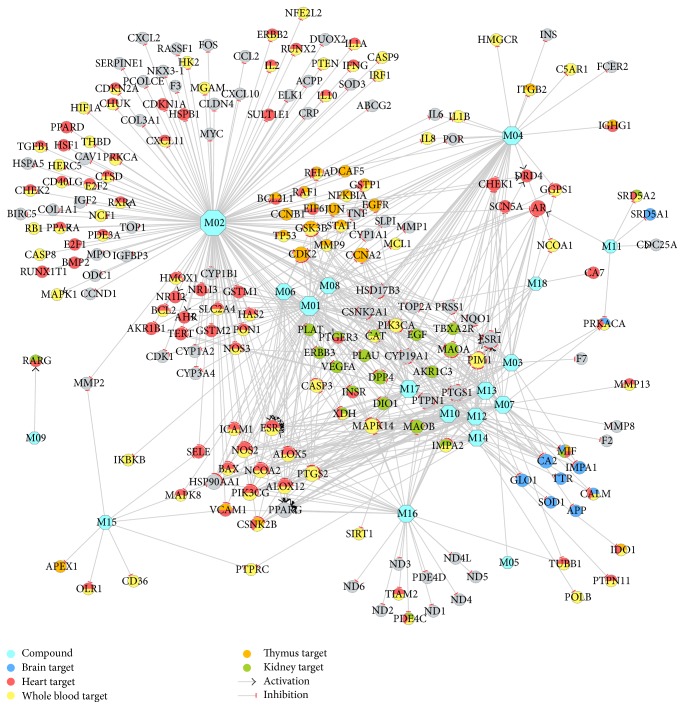
CVDs therapeutic compound-target network. A compound node and a protein node are linked if the protein is targeted by the corresponding compound. Node size is proportional to its degree. The letters are target labels. Arrows indicate activation and T-arrows represent inhibition.

595 D-T interactions (Table S4) data between 18 compounds and 218 targets were generated by the method in Section 2.2. In all the interactions, 35 interactions modulate the 24 (Table 2) brain high-abundant targets through 6 compounds that can freely cross BBB. The 35 interactions were shown in Figure 3, which might explain why Xinnaoxin Pill can be used for the treatment of CBVDs. Other interactions were described in Figure 4 and deemed to decode pharmacological effects for CVDs.

With respect to action modes of the 595 DTIs, 546 are inhibited DTIs, and the others are activated DTIs. Among these DTIs, many of them are identical with those reported in the literature, such as TNF [42] and ESR1 [43]. These results indicate the reliability of the PreAM model.

#### 3.2.1. D-T_1_ Network for Curing CBVDs

Figure 3 shows that there are 24 targets of CBVDs in the C-T_1_ network modulated by 6 compounds that can freely cross the BBB. Among the targets, CA2 (carbonic anhydrase 2) displays the highest degree (DD = 9), followed by MIF (macrophage migration inhibitory factor, DD = 5), GLO1 (lactoylglutathione lyase, DD = 4), IMPA1 (inositol monophosphatase 1, DD = 4), PRKACA (camp-dependent protein kinase catalytic subunit alpha, DD = 3), and so forth. All these results indicate that the three herbs* R. rosea*,* L. chinense*, and* H. rhamnoides* in Xinnaoxin Pill can interact with the cerebrovascular disease-associated proteins. For instance, (1) CA2 plays a critical role in the cerebrovascular disease that triggered Alzheimer's disease [44]. Thus, the inhibition of CA2 by M01 reduces the chances of Alzheimer's disease after cerebrovascular disease; (2) studies suggest that MIF is upregulated in the brain after cerebral ischemia, and disruption of the Mif gene in mice leads to a smaller infarct volume and better sensory-motor function after transient middle cerebral artery occlusion [45]. Similarly, M01 is predicted to inhibit MIF, also responsible for CBVDs; (3) it has been reported that pharmacological inhibition of GLO1 could exacerbate thermal hyperalgesia [46]. Here, we find that molecule M02 (in all three herbs) is able to activate GLO1, thus exerting clinical effect on CBVDs.

Interestingly, the topology of the network does not have a clear bias toward PRKCB (protein kinase C beta type, DD = 2), SPP1 (osteopontin, DD = 2), CALM (calmodulin, DD = 2), and so forth. They are all attractive therapeutic targets curing CBVDs. For example, it has been reported that inhibition of PRKCB increased BBB permeability during hyperglycemic stroke and prevents edema formation* in vivo* [47]. And this target was predicted to be inhibited by M02 in herbs of* R. rosea*,* L. chinense*, and* H. rhamnoides*, further highlighting the efficiency of Xinnaoxin Pill in treatment of CBVDs. SPP1 is a multifunctional protein which has shown neuroprotective properties in animal models of cerebral ischemia [48]. Nevertheless, its role in acute human stroke has not been recognized. Here we found that M15 (vitamin K in* H. rhamnoides*) is capable of inhibiting SPP1, thus elucidating the role of SPP1 in ischemic stroke pathophysiology.

#### 3.2.2. D-T_2_ Network for Combating CVDs

Figure 4 provides a network-level understanding of the mechanism of Xinnaoxin Pill for treating CVDs. It shows that targets (Table 3) with high degree of connectivity (>3), such as ESR1 (estrogen receptor-*α*, DD = 16), ESR2 (estrogen receptor-*β*, DD = 15), AR (androgen receptor, DD = 14), and PIM1 (serine/threonine-protein kinase pim-1, DD = 15), are all therapeutic targets of CVDs. For example, (1) data from animal model and clinical studies support the associations between polymorphisms in both ESRs and CVDs [49, 50]. M02 (quercetin) is shown to block ESRs and AR indicating the effectiveness of the treatment. But unlike M02, M03 (rhodiosin in R. rosea) has active effects on the ESRs. (2) In humans, PIM1 is normally expressed in the cytoplasm of cardiomyocytes in adult myocardial tissue. In fact, study has led to the discovery that PIM1 promotes cardioprotective signaling and enhances cardiac structure and function after pathological injury [51]. In this paper, we found that M03 can inhibit PIM1, which is worth more attention for future studies.

#### 3.2.3. Ligand-Target Analysis

As shown in Table 4, the experimental IC_50_ values from the inhibition test yield an estimate for antigen-antibody reactions, which agree well with those derived from the PreAM model. Compounds of rhodiosin, astragalin, and rutin were tested in PIM1 inhibition assays and proved to be active as inhibitors of it. Although the data highlighted that astragalin (IC_50_ = 62 *μ*M) and rutin (IC_50_ = 57 *μ*M) turned out to be more active than rhodiosin (IC_50_ = 96 *μ*M), indicating that rhodiosin can also repress the vitality of PIM1 as well as astragalin, and rutin. The highly effective compound quercetin bound to PTGS1 with an IC_50_ of 82 *μ*M in a manner characteristic of antagonists. To further define its mode of action, we tested the IC_50_ of compounds of astragalin and kaempferol-3-glucoside. Astragalin with an apparent IC_50_ of about 43 *μ*M had obvious inhibitory effect on the target PTGS1. Interestingly, kaempferol-3-glucoside (IC_50_ = 74 *μ*M) also showed the suppression effect of PTGS1. These findings indicated that the PreAM model can be used accurately and conveniently in assessing the action mode of drug-target interactions.

### 3.3. Multipathways Regulations for Disease Treatment

**Figure 5:**
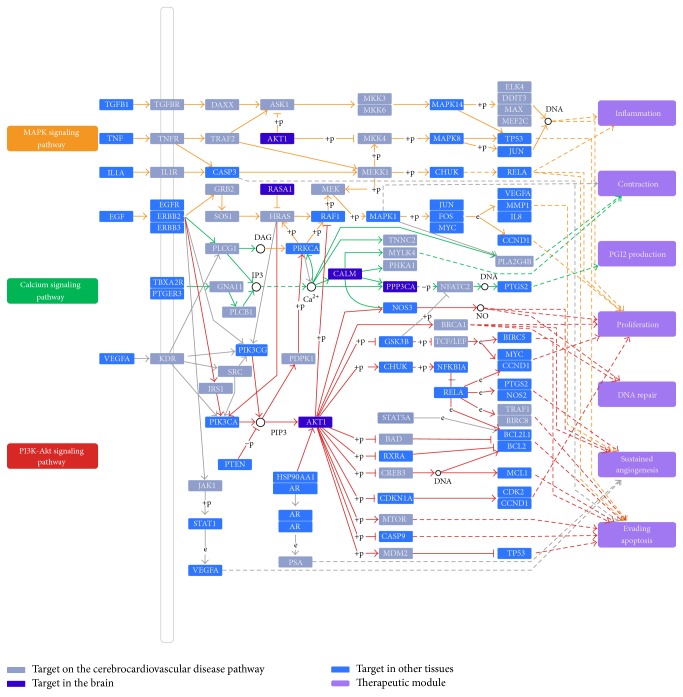
Cerebro-CVD pathway and therapeutic modules.

An incorporated “CCDs pathway” was assembled based on the current knowledge of CCDs pathology. Of the 218 target proteins, 210 can be mapped onto the pathway. They display extremely significantly close functional linkage correlation to the CCDs pathway related proteins (ultimate nearness = 0.034, nearness = 0.008,* P* ≪ 0.01). As shown in Figure 5, the CCDs pathway can be divided into several therapeutic modules, such as inflammation, contraction, PGI2 production, proliferation, DNA repair, sustained angiogenesis, and evading apoptosis. As an illustration, three representative therapeutic modules were described in detail to clarify its therapeutic mechanism.

#### 3.3.1. Inflammation Module

Some targets marked in the MAPK signaling pathway are involved in inflammation process, indicating that the anti-inflammatory action is important for the treatment of CCDs. As shown in Figure 5, M02 can activate and inhibit protein TGFB1 and TNF, respectively. In fact, TGFB1 is a multifunctional cytokine involved in the regulation and proliferation of cells. Cytokine promotes differentiation of leucocytes but has inhibitory effects on proliferation of T lymphocytes and activation of macrophages, suggesting a regulatory role in inflammatory states [52]. TNF promotes inflammation by stimulation of capillary endothelial cell proinflammatory responses and thereby provides leukocyte adhesion and infiltration into the ischemic brain. All this suggests that agents that suppress TNF's production or actions will reduce leukocyte infiltration into ischemic brain regions and thereby diminish the extent of tissue loss [53].

#### 3.3.2. Cardiac Contractility-Associated Module

Calcium signaling pathway might be perturbed by herbal ingredients and produce contraction function, as displayed in Figure 5. For example, ligand M02 is shown with a reduction of PRKCA protein levels and an augmentation of ERBB2, respectively. PRKCA is a fundamental regulator of cardiac contractility and Ca^2+^ handling in myocytes. Research suggests that hypercontractility caused by* Prkca* deletion protects against heart failure induced by pressure overload [54]. ERBB2 is essential in the prevention of dilated cardiomyopathy, which is required for maintenance of cardiac contractility [55]. In addition, M12 serves as an activator of CALM. It is shown that CALM is intrinsically dependent on changes in intracellular Ca^2+^ concentration, which regulates cardiac contractility [56]. All these indicate that Xinnaoxin Pill treats the CCDs through one way of promoting cardiac contractility.

#### 3.3.3. Sustained Angiogenesis-Associated Module

Inadequate blood supply to the heart resulting from insufficient angiogenesis is a hallmark feature of many CVDs. In this situation, promoting angiogenesis is an effective therapeutic program both in the repair process of damaged tissue and in the formation of collateral response to tissue ischemia. As marked in Figure 5, some targets involved in sustained angiogenesis are modulated by the herbal drug through acting on PI3K-Akt signaling pathway. M08 and M02 were observed to increase PTGS2 and NOS3 level, respectively. In addition, M10 was also shown with an increment of NOS2. Recent evidence demonstrates that the inhibition of PTGS2 leads to restricted angiogenesis and downregulates production of proangiogenic factors [57]. NOS3 is a mediator of angiogenesis due to the production of NO, which has been shown to modulate angiogenesis* in vitro* and* in vivo* [58]. Another angiogenesis modulator is NOS2, which appears to exert a direct effect on several angiogenic factors such as VEGF [59]. As stated, angiogenesis is a complex process where several proteins converge and the angiogenesis-based treatment protocol for CCDs that target these proteins will have modest yields and satisfactory results.

### 3.4. Multiple-Target/Organ Cooperation for Disease Treatment

#### 3.4.1. Function Cooperation

**Figure 6:**
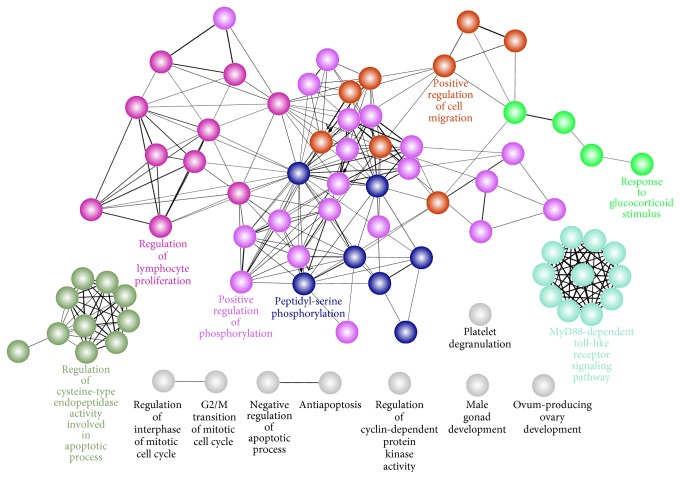
Functional grouped network for the targets of Xinnaoxin Pill. Functionally related groups partially overlap, only the label of the most significant term per group is displayed.

GOBP analysis shows that these targets were enriched to regulate the following biological processes: positive regulation of phosphorylation, MyD88-dependent toll-like receptor signaling pathway, platelet degranulation, positive regulation of cell migration, peptidyl-serine phosphorylation, and so forth (Figure 6). These processes take on different functionality and the mutual cooperation with each other yields an effective therapy for complex diseases.

For example, (1) phosphorylation was the most important protein posttranslational modification which is involved in the regulation of diverse biological processes [60], such as endothelial dysfunction [61]. Dysfunction of NOS3 is implicated as a contributing factor in these varied disease states [62]. (2) Toll-like receptors (TLRs) are differently expressed by vessels within normal human vasculature. Atherosclerosis development in murine models has largely been shown to be associated with increased expression of TLR-2 and TLR-4 [63]. (3) Platelets play a pivotal role in thromboembolism, and a better understanding of the cellular mechanisms involved in platelet activation in ischaemic cerebrovascular disease should improve our understanding of the pathogenesis of stroke and provide opportunities for improved secondary prevention [64]. (4) Cell motility is an important part of many physiological processes such as angiogenesis. Endothelial cell migration and proliferation are central to the process of new blood vessel formation [65].

#### 3.4.2. Target Tissue Location

Microarray analyses of mRNA expression showed that, among the 218 targets, 211 of them had their expression profiles in 84 normal tissues (Table S5). According to BioGPS database, the 211 targets are ubiquitous in human tissues at differing levels. And importantly, we compared expression patterns across different tissues and observed that 24 targets in Table 2 contain higher mRNA expression in brain than the average value of 84 tissues for each target. The 24 brain high-abundant targets are considered as therapeutic target for CBVDs and other low-abundant or hardly expressed 194 targets are thought to be associated with CVDs.

**Figure 7:**
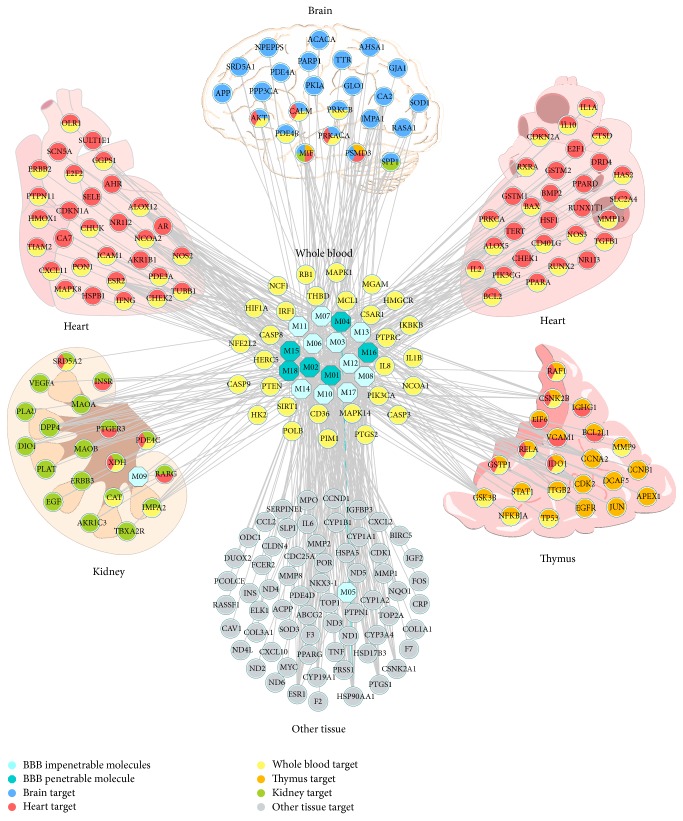
Target organ location map. The node pie chart represents the organs in which each target is located.

Figure 7 shows the tissue distribution network of the 211 targets based on their expression patterns. The networks are divided into a few tissue modules, including brain, heart, kidney, thymus, and whole blood. Meanwhile, the targets in whole blood are linked with tissues in any form. Our results imply that these tissues are closely related to CCDs and the whole blood guides as a bridge of these tissues. For example, many of the strokes involved areas of the brain involved in motor control, such as the basal ganglia [66]. Heart rate has been shown to be an important predictor of mortality in CVDs [67]. Cardiovascular morbidity and mortality in patients with chronic kidney disease (CKD) are high, and the presence of CKD worsens outcomes of CVDs [68]. Patients with thymus hypoplasia have a very high incidence of congenital heart disease [69].

## 4. Discussion and Conclusion

Medicine is undergoing a revolution that will transform the practice of healthcare in virtually every way. This revolution is emerging from the convergence of systems pharmacology—a systems approach to medicine. Holistic medicine is the art and science of healing that addresses care of the whole body, especially complex diseases. It promises to (1) define the components of the system, (2) clarify how these components interact with one another, (3) stratify complex diseases into their distinct subtypes for an impedance match against proper drugs, and (4) provide deep insights into disease mechanisms [70].

The workflow for TCM-based holistic medicine constructs an easily-interpretable map that could facilitate understanding how the holistic medicine through systems pharmacology means to remedy chronic conditions from a molecular to systems level, which provides guidance for ascertaining complicated nosogenesis, systems cure strategies, and new drug development.

Generally, the treatment of holistic medicine is chartered as multicomponent therapeutics, multitarget/pathways regulations, and multiorgan cooperation. To define the components of the system, we developed a novel systems pharmacology approach integrating with multiple techniques including ADME-systems evaluation, drug targeting, and target tissue distributing. With the aid of ADME-systems evaluation, 18 active compounds were detected. And these compounds could interact with 218 locations various targets (several times of the compounds) by drug targeting, indicating the multicomponent therapeutics and multitarget regulations characteristic.

To clarify how these components interact with one another, a PreAM model was built to evaluate the action mode between drugs and targets. We find that most drugs have multiple organs support by inhibiting targets. And the drug-pathway analysis shows the characteristic of multitarget/pathways regulations, which result in several therapeutic modules, such as inflammation, contraction, and sustained angiogenesis. These results not only mirror the treatment feature of holistic medicine but also promote the development of new drugs. In addition, it points to the way for the treatment of complex diseases.

Complex diseases largely arise from an integration of genetic, environmental, and lifestyle factors. Yet, unlike the Mendelian diseases, which are caused by abnormalities to a single gene, complex diseases are chiefly unknown due to byzantine genetic and organic interactions. To treat chronic disease, there are several means in allopathic medicine such as cocktails of drugs and drug combination. But they are always weakly effective. In holistic medicine, the cardiovascular and cerebrovascular diseases were stratified into their distinct subtypes (CVDs and CBVDs) for an impedance match against proper drugs. Molecules with BBB permeability directly activate or block the targets located in brain and further boost the functional recovery after CBVDs by suppressing the complications, such as Alzheimer's disease and stroke pain. Compounds interact with nonbrain location targets such as sex hormone receptors that are effective in treating CVDs (hypertension, coronary artery disease, etc.).

As exemplified by CCDs, the present study demonstrates the effectiveness of holistic medicine in curing complex diseases from a molecular to system level and importantly provides a paradigm for the treatment of complex diseases in the near future.

## Supplementary Material

Table S1 shows the structure information of 367 compounds in Xinnaoxin Pill. Table S2 and Table S3 respectively represents the information for construction PreHIA and PreAM model. Table S4 displays the detailed information of compound-target interactions. And Table S5 shows the information of the 218 targets in this study. Figure S1 and Figure S2 reveal the workflow for PreHIA and PreAM.

## Figures and Tables

**Table 1:** Active compounds in Xinnaoxin Pill with corresponding pharmacokinetics parameters.

Number	Molecular name	Structure	OB	DL	HL	HIA	BBB	Herb
M01	Kaempferol	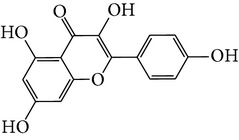	42.3	0.24	14.71	<70%	Y	*R. rosea*; *L. chinense*; *H. rhamnoides *

M02	Quercetin	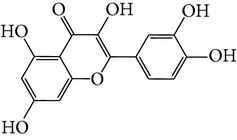	42.41	0.28	14.22	<70%	Y	*R. rosea*; *L. chinense*; *H. rhamnoides *

M03	Rhodiosin_DG^*∗*^	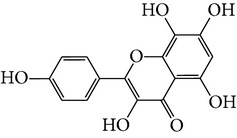	37.86	0.27	14.72	<70%	N	*R. rosea *

M04	Rutin_DG^*∗*^	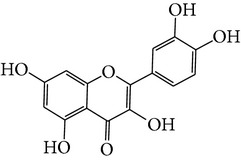	47.05	0.28	14.23	<70%	Y	*R. rosea*; *H. rhamnoides *

M05	(24R)-4alpha-Methyl-24-ethylcholesta-7,25-dien-3beta-yl acetate	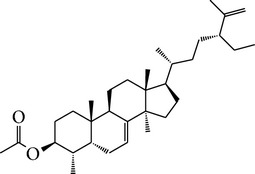	46.36	0.84	8.25	≥70%	Y	*L. chinense *

M06	7-O-Methyl luteolin-6-C-beta-glucoside_DG^*∗*^	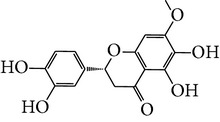	44.39	0.3	14.13	<70%	N	*L. chinense *

M07	Glycitein	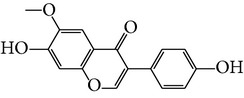	50.48	0.24	16.32	≥70%	N	*L. chinense *

M08	Physcion-8-O-beta-D-gentiobioside_DG^*∗*^	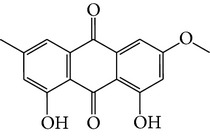	80.77	0.27	30.98	<70%	N	*L. chinense *

M09	Mutatoxanthin	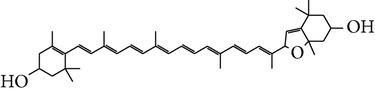	38.38	0.59	16.45	≥70%	N	*L. chinense *

M10	Astragalin_DG^*∗*^	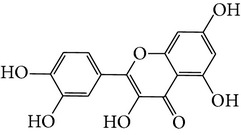	64.34	0.24	14.74	<70%	N	*H. rhamnoides *

M11	Cholesteryl benzoate	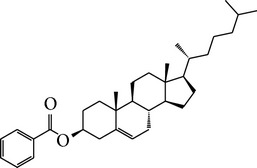	48.08	0.84	8.12	≥70%	N	*H. rhamnoides *

M12	Isorhamnetin-3-O-*β*-rutinoside_DG^*∗*^	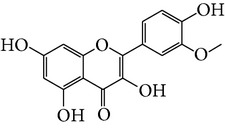	50.13	0.31	14.35	<70%	N	*H. rhamnoides *

M13	Kaempferol-3-glucoside_DG^*∗*^	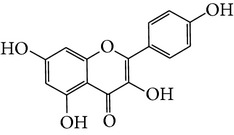	41.91	0.24	14.75	<70%	N	*H. rhamnoides *

M14	Phosphatide	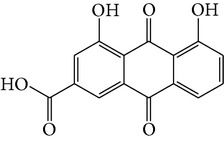	31.81	0.28	30.93	<70%	N	*H. rhamnoides *

M15	Vitamin K	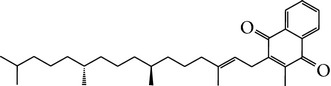	47.6	0.65	28.4	≥70%	Y	*H. rhamnoides *

M16	Vitamin P_DG^*∗*^	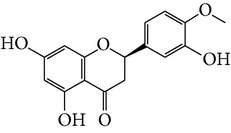	48.07	0.27	15.62	<70%	Y	*H. rhamnoides *

M17	Qercetin-3-O-galactoside_DG^*∗*^	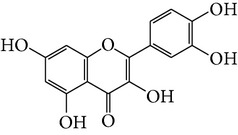	44.61	0.28	14.36	<70%	N	*H. rhamnoides *

M18	(−)-Epicatechin	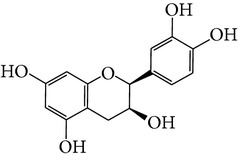	35.78	0.77	16.33	<70%	Y	*H. rhamnoides *

*∗* represents the compounds that have been deglycosylated (DG); Y: Yes it is crossing BBB; N: Not crossing BBB.

**Table 2:** The CBVDs target information.

Gene name	Protein name	Degree
CA2	Carbonic anhydrase 2	4
GLO1	Lactoylglutathione lyase	3
MIF	Macrophage migration inhibitory factor	3
AHSA1	Activator of 90 kDa heat shock protein ATPase homolog 1	2
AKT1	RAC-alpha serine/threonine-protein kinase	2
CALM	Calmodulin	2
PRKCB	Protein kinase C beta type	2
PSMD3	26S proteasome non-ATPase regulatory subunit 3	2
SOD1	Superoxide dismutase [Cu-Zn]	2
SPP1	Osteopontin	2
ACACA	Acetyl-CoA carboxylase 1	1
GJA1	Gap junction alpha-1 protein	1
IMPA1	Inositol monophosphatase	1
NPEPPS	Puromycin-sensitive aminopeptidase	1
PARP1	Poly [ADP-ribose] polymerase 1	1
PDE4A	cAMP-specific 3′,5′-cyclic phosphodiesterase 4A	1
PDE4B	cAMP-specific 3′,5′-cyclic phosphodiesterase 4B	1
PKIA	cAMP-dependent protein kinase inhibitor alpha	1
PPP3CA	Serine/threonine-protein phosphatase 2B catalytic subunit alpha isoform	1
PRKACA	cAMP-dependent protein kinase catalytic subunit alpha	1
RASA1	Ras GTPase-activating protein 1	1

**Table 3:** The CVDs target information.

Gene name	Protein name	Degree
ESR1	Estrogen receptor	15
ESR2	Estrogen receptor beta	14
PIM1	Proto-oncogene serine/threonine-protein kinase Pim-1	14
AR	Androgen receptor	13
HSP90AA1	Heat shock protein HSP 90-alpha	12
MAPK14	Mitogen-activated protein kinase 14	12
NOS2	Nitric oxide synthase, inducible	12
PIK3CG	Phosphatidylinositol-4,5-bisphosphate 3-kinase catalytic subunit gamma isoform	12
PPARG	Peroxisome proliferator-activated receptor gamma	12
PTGS1	Prostaglandin G/H synthase 1	12
PTGS2	Prostaglandin G/H synthase 2	12
PTPN1	Tyrosine-protein phosphatase non-receptor type 1	12
CDK2	Cell division protein kinase 2	11
GSK3B	Glycogen synthase kinase-3 beta	11
ALOX12	Arachidonate 12-lipoxygenase, 12S-type	10
CCNA2	Cyclin-A2	10
ALOX5	Arachidonate 5-lipoxygenase	9
CA2	Carbonic anhydrase 2	9
MAOA	Amine oxidase [flavin-containing] A	9
NCOA2	Nuclear receptor coactivator 2	9
CSNK2A1	Casein kinase II subunit alpha	7
CSNK2B	Casein kinase II subunit beta	7
CYP19A1	Cytochrome P450 19A1	7
DPP4	Dipeptidyl peptidase 4	7
HSD17B3	Estradiol 17-beta-dehydrogenase 3	7
PIK3CA	Phosphatidylinositol-4,5-bisphosphate 3-kinase catalytic subunit alpha isoform	7
CHEK1	Serine/threonine-protein kinase Chk1	6
MAOB	Amine oxidase [flavin-containing] B	6
CASP3	Caspase-3	5
IMPA2	Inositol monophosphatase 2	5
MIF	Macrophage migration inhibitory factor	5
PRSS1	Trypsin-1	5
BAX	Apoptosis regulator BAX	4
GLO1	Lactoylglutathione lyase	4
IMPA1	Inositol monophosphatase	4
TOP2A	DNA topoisomerase 2-alpha	4
VCAM1	Vascular cell adhesion protein 1	4
AKR1B1	Aldose reductase	3
CAT	Catalase	3

**Table 4:** IC_50_ values for the randomly selected drug-target interactions.

Number	Target gene name	Drug name	IC_50_ (*μ*M): mean ± SD
1	PIM1	Rhodiosin	96 ± 3.2
2	PIM1	Astragalin	62 ± 9.7
3	PIM1	Rutin	57 ± 5.8
4	PTGS1	Quercetin	464 ± 6.4
5	PTGS1	Astragalin	143 ± 7.0
6	PTGS1	Kaempferol-3-glucoside	181 ± 11.5
