# Supplementary figures and images for: Systems Pharmacology Dissecting Holistic Medicine for Treatment of Complex Diseases: An Example Using Cardiocerebrovascular Diseases Treated by TCM
Source: Evid Based Complement Alternat Med. 2015 May 26;2015:980190. doi: 10.1155/2015/980190 (PMC4460250; doi:10.1155/2015/980190)

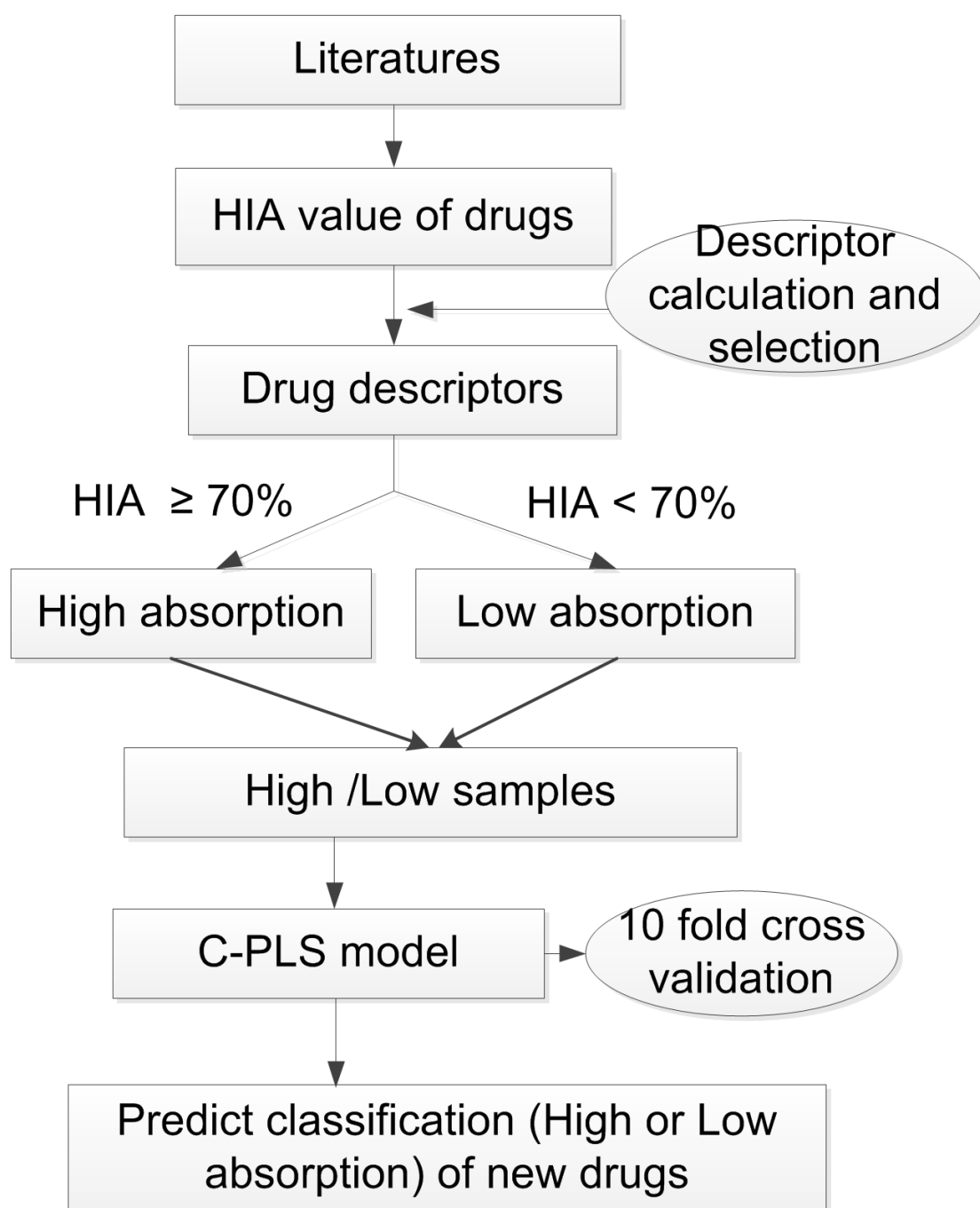

Figure S1. Workflow for PreHIA.

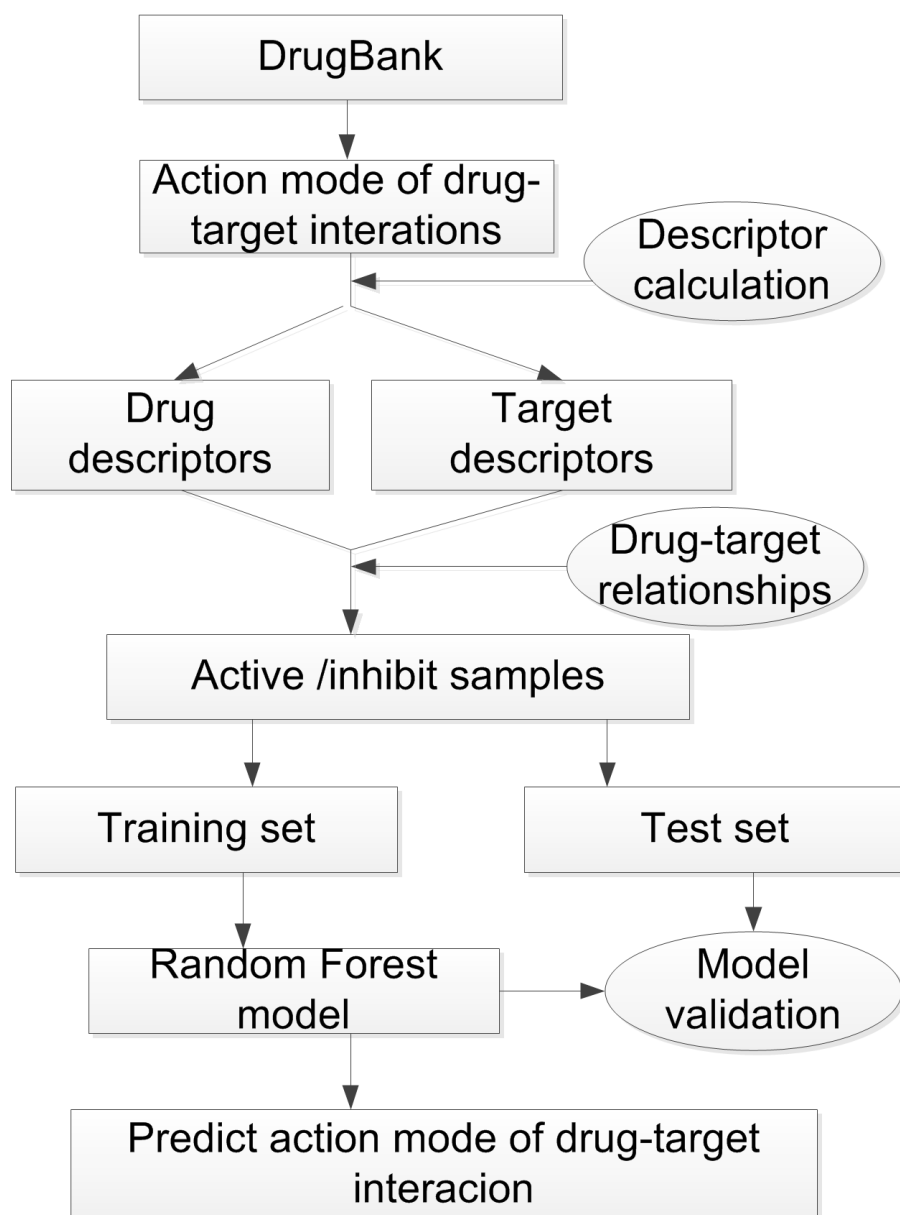

Figure S2. Workflow for PreAM.

Supplement: Supplementary file 1 — Table S1 shows the structure information of 367 compounds in Xinnaoxin Pill. Table S2 and Table S3 respectively represents the information for construction PreHIA and PreAM model. Table S4 displays the detailed information of compound-target interactions. And Table S5 shows the information of the 218 targets in this study. Figure S1 and Figure S2 reveal the workflow for PreHIA and PreAM. [file 980190.f1.zip › mat.980190.v2/980190 suppl. figs.pdf]
